# The crucial role of cardiac MRI parameters in the prediction of outcomes in acute clinically suspected myocarditis: A functional and feature-tracking study

**DOI:** 10.3389/fcvm.2022.946435

**Published:** 2022-09-07

**Authors:** Marzieh Motevalli, Sanaz Asadian, Foroogh Khademi, Nahid Rezaeian, Leila Shayan

**Affiliations:** ^1^Rajaie Cardiovascular Medical and Research Center, Iran University of Medical Sciences, Tehran, Iran; ^2^Trauma Research Center, Rajaee (Emtiaz) Trauma Hospital, Shiraz University of Medical Sciences, Shiraz, Iran

**Keywords:** acute myocarditis, major adverse cardiovascular events (MACE), cardiac MRI, feature tracking (FT), myocardial strain

## Abstract

**Background:**

The definitive diagnosis of myocarditis is made by endomyocardial biopsy, but it is an invasive method. Recent investigations have proposed that cardiac MRI parameters have both diagnostic and prognostic roles in assessing myocarditis. We aimed to evaluate the role of functional and feature-tracking (FT)-derived strain values in predicting major adverse cardiovascular events (MACE) in patients with acute myocarditis.

**Methods and results:**

We evaluated 133 patients with acute myocarditis (74.4% men) between January 2016 and February 2021. During a mean follow-up of 31 ± 16 months, sixteen patients (12.03%) experienced MACE: three deaths (2.3%), nine ICD implantations (6.76%), and five cardiac transplantations (3.8%). The left ventricular ejection fraction (LVEF), the LV end-diastolic volume index (EDVI), and the LV global longitudinal strain (GLS) were the strongest predictors of MACE. Each 1-unit decline in LVEF and LVGLS or 1-unit rise in LVEDVI resulted in a 5, 24, and 2% increase in MACE, respectively. LVEF ≤36.46% and LVGLS ≤9% indicated MACE with 75% sensitivity and 74.4 and 73.5% specificity, respectively.

**Conclusions:**

In a group of acute myocarditis patients with evidence of myocardial edema and late Gadolinium enhancement, LVEF and GLS were the strongest predictors of adverse cardiac events.

## Background

Myocarditis is an inflammatory myocardial disorder diagnosed by clinical, imaging, histological, immunological, and immunohistochemical criteria. Myocardial involvement is typically due to systemic viral infections, consisting of systemic inflammatory diseases and toxins; nonetheless, various infectious and non-infectious causes can result in myocarditis (1, 2).

It is difficult to ascertain the exact frequency of myocarditis due to the wide variety of clinical signs and symptoms. It is assumed that its incidence is 1–10 cases per 100 000 persons. Clinical history ranges from mild symptoms to findings of acute decompensation of heart failure. Feldman et al. classified myocarditis as follows: fulminant myocarditis, acute myocarditis, chronic active myocarditis, and chronic persistent myocarditis (3). Endomyocardial biopsy confers a definitive diagnosis of myocarditis, but it has considerable drawbacks. It is an invasive method and is prone to sampling error (4). The Lake Louise criteria (LLC) in cardiac magnetic resonance (CMR) examination consist of myocardial edema, hyperemia, and late gadolinium enhancement (LGE), and the updated LLC with mapping techniques, which constitute the cornerstone for diagnosing myocarditis, obviates the requirement for endomyocardial biopsy (2, 5).

Although the cardiac condition of most patients with myocarditis improves over time, some patients progress to devastating consequences. Accordingly, finding measures to predict prognosis is of utmost importance. Various CMR studies have derived different results on the predictive role of CMR parameters in patients with myocarditis (6, 7).

Strong evidence characterizes the role of echocardiographic strain especially global longitudinal strain (GLS) in evaluating different myocardial disorders (8, 9).

Recent research has underscored the value of CMR-derived myocardial strain measurement in the assessment of different types of cardiomyopathies (10–12). Evaluation of traditional CMR markers of the ejection fraction (EF) and LGE has a prognostic effect; nevertheless, little is known about the role of novel CMR methods, including feature tracking (FT) and mapping values (13).

Some previous studies have shown a significant association between cardiac prognosis and decreased ventricular strain values in myocarditis and other cardiomyopathies (6, 14–16).

In the present study, we aimed to evaluate the role of functional and FT-derived CMR strain values in predicting major adverse cardiovascular events (MACE) in patients with the diagnosis of acute myocarditis.

## Methods

### Study design and population

**Figure 1:**
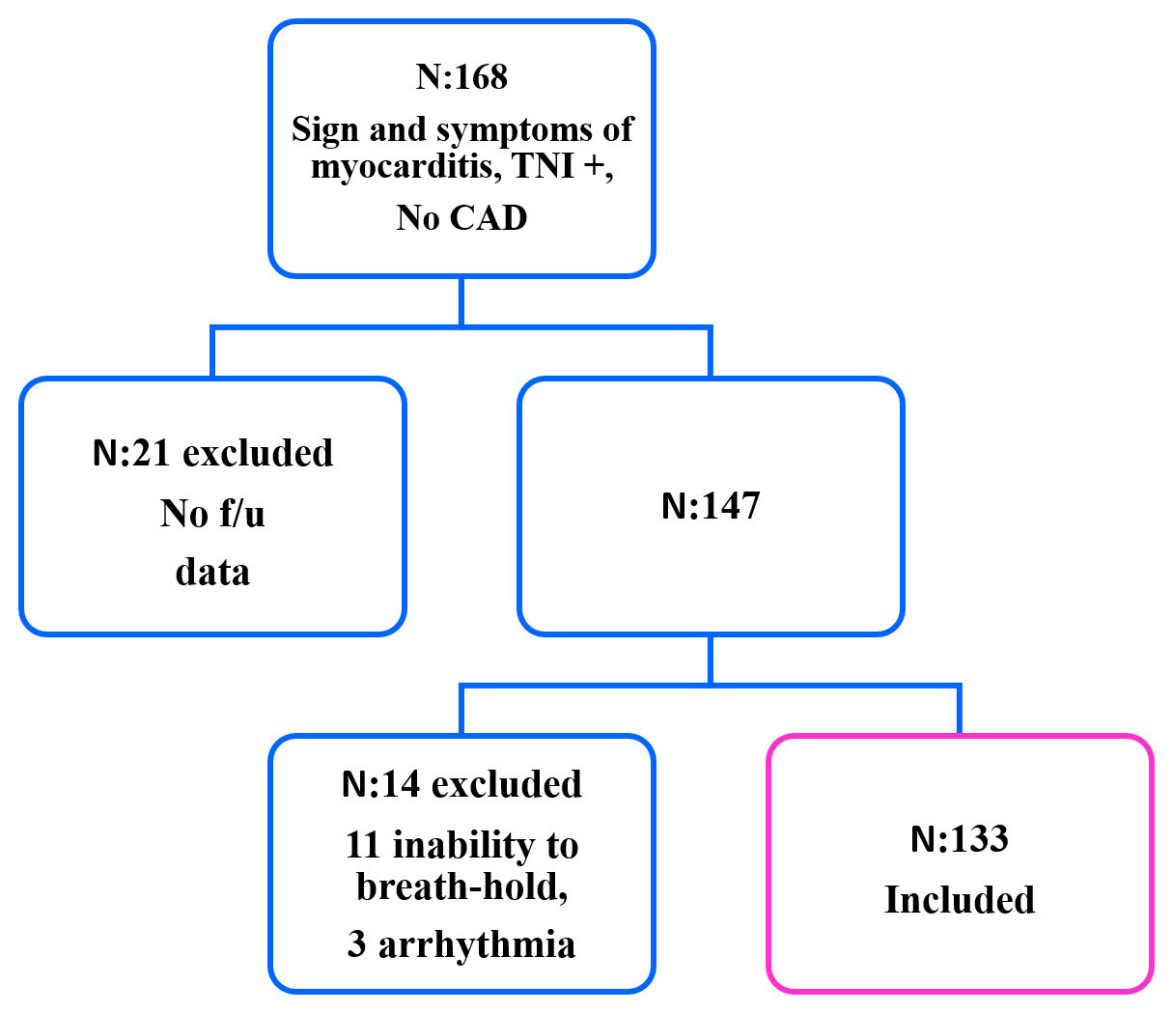
Flowchart of myocarditis patient selection. •f/u, follow-up; TNI, troponin I; CAD, coronary artery disease.

We evaluated all CMR examinations with a final diagnosis of acute myocarditis between January 2016 and February 2021. Based on electronic reports, the selection criteria were among patients who had all these four criteria: (1) History of acute symptoms (including dyspnea and chest pain) within a few days before admission. (2) Increased troponin levels. (3) Normal coronary angiography (no coronary artery disease) or CT angiography results during hospitalization. (4) CMR images with evidence of myocardial edema as well as mid-wall and subepicardial LGE (17). We found 168 CMR reports compatible with these myocarditis criteria. Among these reports, in 21 patients, the follow-up data was unavailable; in 14 subjects, the image quality was not acceptable (significant image artifact due to the patient's inability to breathe-hold in 11 patients and arrhythmia in three patients). Finally, 133 patients were selected with a diagnosis of acute myocarditis based on mentioned criteria (Figure 1). The exclusion criteria comprised the presence of a single criterion of myocardial edema or only being LGE + in the CMR study, subendocardial pattern of myocardial fibrosis, more-than-mild valvular disease, other cardiac pathologies including cardiomyopathy, and ischemic heart disease. An expert cardiologist with at least 6 years of experience in cardiac imaging conducted all measurements.

MACE was defined as the incidence of cardiac death, an implantable cardioverter-defibrillator (ICD) implantation, or heart transplantation.

### Follow-up

All the patients were assessed for the possible existence of MACE during a follow-up time of 10–69 months (mean ± SD: 31 ± 16 mon). We extract the follow-up data from patients' electronic records and telephone calls.

### CMR examination

CMR was accomplished using a 1.5-Tesla scanner (Siemens Avanto, Erlangen, Germany) with an 8-element cardiac-phased array receiver surface coil.

### CMR images

All ECG-gated cine functional sequences were done during an end-expiratory breath-hold. Left ventricular (LV) 2, 3, and 4-chamber views, as well as a stack of contiguous short-axis with LV coverage from inflow to apex, were acquired with sequence parameters, including a field of view (FOV) of 300 mm, an imaging matrix of 156 × 192, a slice thickness of 8 mm, no interslice gaps for short-axis images, and repetition time/ echo time of 31/1.2 ms. LV volume and LV systolic function were analyzed by tracing end-diastolic and end-systolic endocardial borders on cine short-axis images.

**Figure 2:**
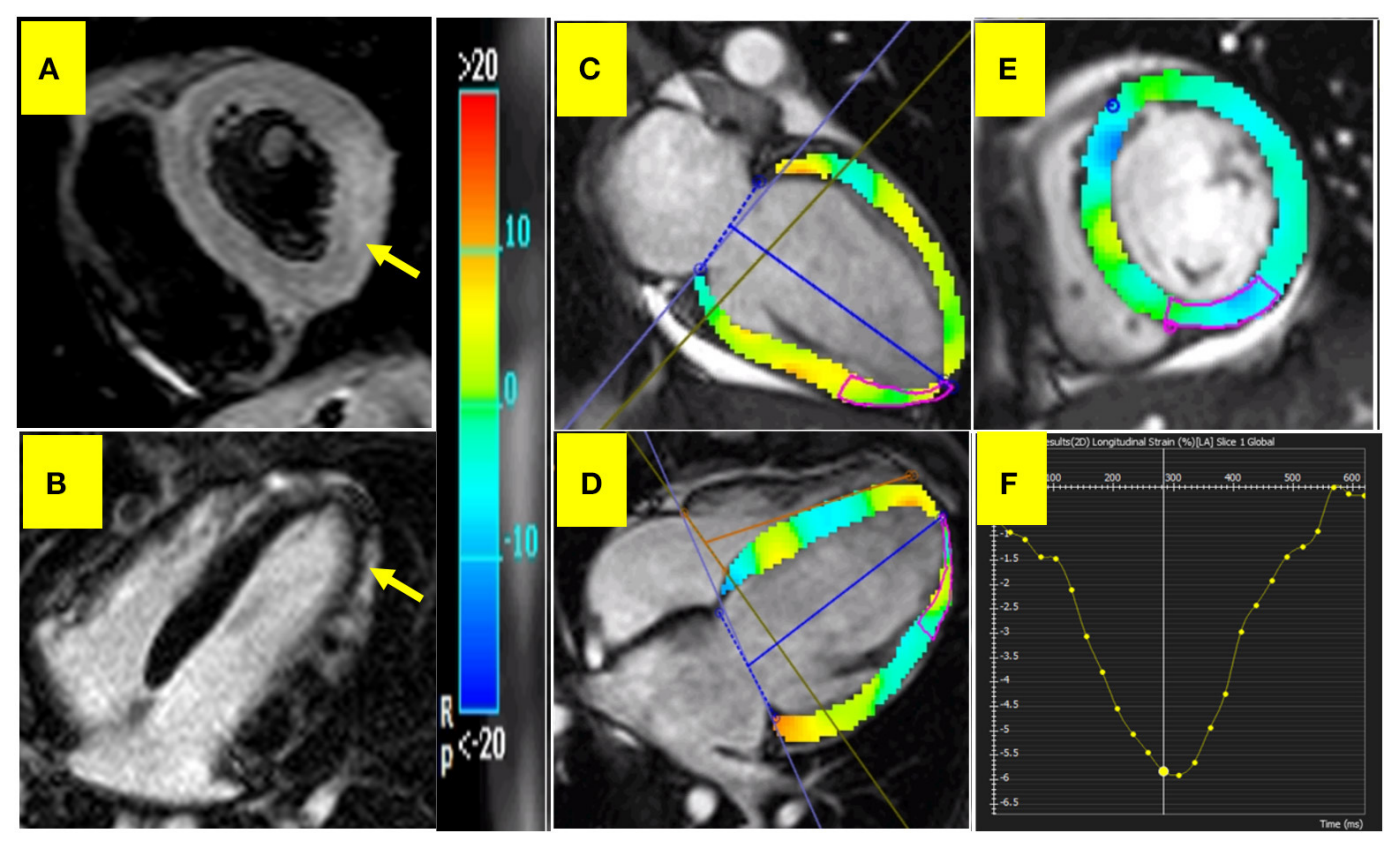
CMR in acute myocarditis. **(A)** myocardial inflammation. **(B)** Subepicardial LGE in LV inferolateral wall. **(C–E)** Depict feature tracking myocardial strain in 2, 4-chamber, and short-axis views, respectively. **(F)** Depicts longitudinal strain curve.

Myocardial inflammation was assessed utilizing the short tau inversion recovery (STIR) series as a ratio of myocardial-to-skeletal muscle signal intensity of more than 1.9 (Figure 2A).

LGE extent was determined visually by a 17-segment model; the presence and absence of LGE in each LV myocardial segment were scored as 1 and 0, respectively, and the sum of the scores (maximum: 17) was reported as the LGE-visual presence score (LGE-VPS) (18).

Due to the retrospective nature of our study, the mapping technique was unavailable. Therefore, we selected the myocarditis patients according to the old LLC, including 2 of 3 positive findings of myocardial hyperemia, edema, and LGE (Figure 2B).

### Feature-tracking CMR method

CMR images were analyzed offline utilizing the FT software CVI 42 (Circle Cardiovascular Imaging, Calgary, Alberta, Canada), version 5.6.2 (634). All endocardial and epicardial borders were manually traced in the end-diastolic phase and then propagated to the whole cardiac cycle in short-axis stacks and 2-, 3-, and 4-chamber planes to identify 3D global longitudinal, circumferential, and radial strains (GLS, GCS, and GRS, respectively). Additionally, 2D right ventricular GLS was extracted from the 4-chamber view and RVGCS and RVGRS from the short-axis view. Image brightness was adjusted to discriminate between the endocardium and the blood pool. Longitudinal strain depicts longitudinal myocardial fiber shortening from the base to the apex, while circumferential strain shows the percentage of myocardial shortening around the perimeter (Figures 2C–F). On the other hand, radial strain denotes the percentage of myocardial wall thickening. All the strain values were expressed as absolute values.

### Myocardial biopsy

All patient's electronic medical records were evaluated for myocardial biopsy data.

### Statistical analysis

The SPSS software, version 22.00, was utilized for analyses. Continuous variables were presented as the mean ± SD, while categorical variables were demonstrated as frequencies and percentages. Considering the sample size discrepancy between the MACE positive and MACE negative groups, we applied the Mann–Whitney *U* test to compare the parameters between the 2 groups. Independent sample *t*-test was to compare CMR parameters between groups with LVEF≥ 40 and <40%. Significantly different variables were entered in the stepwise logistic regression analysis to find the significant variables in the regression model to reveal the occurrence of MACE. The receiver operating characteristic (ROC) analysis was utilized to find the cutoff values of CMR parameters for predicting MACE. The area under the curve (AUC), sensitivity, and specificity were reported. A cutoff value of 0.05 was considered to denote statistically significant results.

## Results

The study population was composed of 133 patients, including 74.4% men, at a mean ± SD age of 37 ± 16 years diagnosed with myocarditis between January 2016 and February 2021. Table 1 demonstrates the demographic characteristics and CMR parameters of the study participants. All patients had normal coronary CT angiography/angiography results during hospitalization. The mean ± SD interval between CT angiography/angiography and CMR was: 3 ± 1.41 days. All CMR parameters were compared between groups with LVEF ≥40% and LVEF <40%. The results are depicted in Table 2.

**Table 1:** Demographic and baseline CMR parameters of the study population.

**Variables**	Frequency (*n*)*	**Percent %**
Gender (male)	99	74.4
Cardiac death	3	2.3
ICD	9	6.76
Cardiac transplantation	5	3.8
**Variables**	**Mean**	**SD**
AGE (year)	36.5	16
LVEF (%)	41	14
RVEF (%)	46	13
LA volume (ml)	65	37
RA volume (ml)	51	47
LVEDVI (ml/m^2^)	94	48
RVEDVI (ml/m^2^)	83	31
LVGLS %	11.5	4.3
LVGCS %	13.5	5.1
LVGRS %	27.7	13.9
RVGLS %	19.6	7.2
RVGCS %	8.6	4
RVGRS %	14.3	7.9
LSSR 1/sec	−0.78	0.88
CSSR 1/sec	−0.88	0.51
RSSR	+1.9	1.2
LDSR	+0.89	1.8
CDSR	+0.86	0.38
RDSR	−1.86	1.17

* n, number; SD, standard deviation; LV, left ventricle; ICD, implantable cardioverter defibrillator; RV, right ventricle; LV, left ventricle; EF, ejection fraction; LA, left atrium; RA, right atrium; EDVI, end-diastolic volume index; ESVI, end-systolic volume index.

GLS, global longitudinal strain; GCS, global circumferential strain.

GRS, global radial strain; SV, stroke volume; LSSR, longitudinal systolic strain rate; CSSR, Circumferential systolic strain rate; RSSR, LSSR; Radial systolic strain rate; LDSR, longitudinal diastolic strain rate; CDSR, Circumferential diastolic strain rate; RDSR, Radial systolic strain.

**Table 2:** Comparisons of CMR parameters between LVEF groups.

	**LVEF≥40%** ***n*: 81**	**LVEF<40%** ***n*: 52**	
**Variables**	**Frequency (** ***n*** **)/ percent %**	**Frequency (** ***n*** **)/ percent %**	***P*** **-value**
Gender (male)	63 (77.8)	36 (69.2)	0.3
MACE	4 (4.94)	12 (23.08)	0.002
Cardiac death	1 (1.23)	2 (3.85)	0.56
ICD	2 (2.5)	7 (13.5)	0.02
Cardiac transplantation	1 (1.23)	4 (7.7)	0.07
**Variables**	**Mean** **±** **SD**	**Mean** **±** **SD**	
AGE (year)	33.8 ± 14.49	40.7 ± 17.7	0.01
LVEF (%)			
RVEF (%)	51.07 ± 7.99	39.1 ± 15.8	<0.001
LA volume (ml)	55.91 ± 27.09	79.6 ± 45.2	0.001
RA volume (ml)	44.67 ± 22.07	59.6 ± 69.9	0.14
LVEDVI (ml/m^2^)	82.68 ± 28.0	112.2 ± 64.4	0.003
RVEDVI (ml/m^2^)	79.31 ± 26.1	89.17 ± 37.62	0.1
LGE-VPS	2.44 ± 1.37	2.7 ± 2.4	0.4
LVGLS %	13.87 ± 3.1	7.9 ± 3.24	<0.001
LVGCS %	16.3 ± 3.3	9.21 ± 4.25	<0.001
LVGRS %	34.7 ± 12.0	16.84 ± 8.84	
RVGLS %	21.4 ± 6.3	16.7 ± 7.53	<0.001
RVGCS %	10.0 ± 3.63	6.41 ± 3.83	<0.001
RVGRS %	16.4 ± 6.8	10.89 ± 8.35	<0.001
LSSR 1/sec	−0.91 ± 1.08	−0.59 ± 0.28	0.03
CSSR 1/sec	−0.99 ± 0.54	−0.70 ± 0.42	0.001
RSSR	2.2 ± 1.1	1.20 ± 0.86	<0.001
LDSR	1.0 ± 2.26	0.65 ± 0.43	0.2
CDSR	0.99 ± 0.38	0.66 ± 0.30	<0.001
RDSR	−2.3 ± 1.1	−1.13 ± 0.79	<0.001

n, number; SD, standard deviation; LV, left ventricle; ICD, implantable cardioverter defibrillator; RV, right ventricle; LV, left ventricle; EF, ejection fraction; LA, left atrium; RA, right atrium; EDVI, end-diastolic volume index; ESVI, end-systolic volume index.

LGE-VPS, late gadolinium enhancement-Visual presence score.

GLS, global longitudinal strain; GCS, global circumferential strain.

GRS, global radial strain, SV, stroke volume; LSSR, longitudinal systolic strain rate; CSSR, Circumferential systolic strain rate; RSSR, Radial systolic strain rate; LDSR, longitudinal diastolic strain rate; CDSR, Circumferential diastolic strain rate; RDSR, Radial systolic strain.

Sixteen out of the 133 patients (12.03%) experienced MACE during a mean ± SD follow-up of 31 ± 16 months: three deaths (2.3%), nine ICD implantations (6.76%), and five cardiac transplantations (3.8%). One patient (0.75%) underwent ICD implantation and heart transplantation.

As we can evaluate the previous reports among patients with history of ICD implantation, seven out of nine patients had LVEF< 36%, and two out of nine patients had a history of recurrent VT that resulted in ICD implantation.

The Mann–Whitney *U* test revealed a significant difference in the following parameters in patients with and without MACE: biventricular EF and end-diastolic volume index (EDVI), left atrial (LA) volume, biventricular GLS, LVGCS, LVGRS, LV global longitudinal systolic strain rate and LV global circumferential diastolic strain rate (Table 3).

**Table 3:** Comparison of age and CMR parameters between MACE negative and positive groups.

**Variable**	**Median (IQR) MACE negative N = 117**	**Median (IQR) MACE positive** **N = 16**	***P*-value**
AGE (year)	37 (29)	35.5 (20)	0.2
LVEF (%)	44.14 (16.92)	23.00 (27.71)	<0.001
RVEF (%)	50.47 (13.09)	36.09 (27.54)	0.003
LA volume (ml)	53.00 (40.07)	69.39 (70.62)	0.04
RA volume (ml)	40.00 (30.67)	50.00 (39.32)	0.3
LVEDVI (ml/m^2^)	81.42 (26.95)	108.34 (107.02)	0.01
RVEDVI (ml/m^2^)	77.00 (33.38)	88.40 (67.00)	0.04
LVGLS %	12.00 (6)	7.39 (7)	<0.001
LVGCS %	14.63 (6)	7.24 (10)	0.001
LVGRS %	28.21 (19)	12.95 (23)	0.01
RVGLS %	21.23 (9)	13.06 (12)	0.003
RVGCS %	8.35 (5)	6.64 (8)	0.1
RVGRS %	13.00 (10)	10.12 (17)	0.3
LSSR 1/sec	−0.80 (0.39)	−0.49 (0.36)	0.001
CSSR 1/sec	−0.96 (0.35)	−0.70 (0.68)	0.07
RSSR	1.93 (1.17)	0.97 (2.51)	0.06
LDSR	0.73 (0.45)	0.56 (0.51)	0.1
CDSR	0.87 (0.48)	0.61 (0.50)	0.04
RDSR	−1.77 (1.38)	−1.31 (1.16)	0.1
LGE-VPS	2.00 (3)	2.00 (3)	0.5

SD, standard deviation; IQR, interquartile range; LV, left ventricle; RV, right ventricle; LV, left ventricle; EF, ejection fraction; LA, left atrium; RA, right atrium; EDVI, end-diastolic volume index; ESVI, end-systolic volume index.

GLS, global longitudinal strain; GCS, global circumferential strain.

GRS, global radial strain; SV, stroke volume; LSSR, longitudinal systolic strain rate; CSSR, Circumferential systolic strain rate, RSSR, Radial systolic strain rate, LDSR, longitudinal diastolic strain rate; CDSR, Circumferential diastolic strain rate; RDSR, Radial systolic strain; VPS, Visual presence score.

All significantly different variables were entered in the stepwise logistic regression analysis in the next step. Only LVEF and LVEDVI were meaningful in the model with an odds ratio of 0.946, confidence interval (CI): 0.904–0.989 (*P* = 0.01) and 1.017 and CI: 1.006–1.028 (*P* = 002), respectively. In other words, each 1-unit decrease in LVEF and increase in LVEDVI resulted in a 5 and 2% rise in the incidence of MACE, respectively.

The biventricular deformation parameters were entered in the stepwise logistic regression analysis. LVGLS was the meaningful variable with an odds ratio of 0.760 and CI:0.655–0.882 (*P* < 0.001). That is to say, each 1% decrease in LVGLS resulted in a 24% increase in the incidence of MACE.

**Figure 3:**
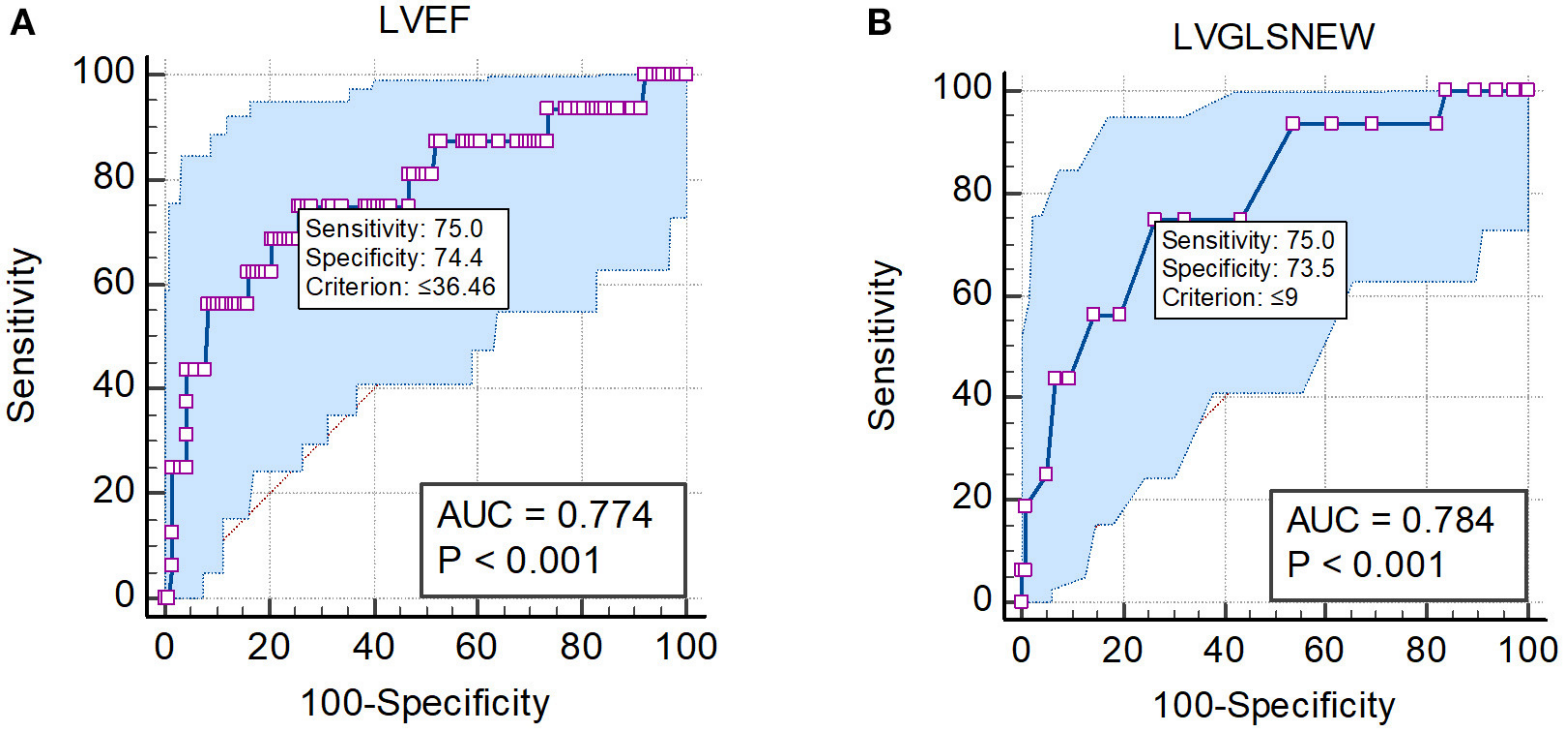
ROC analysis results for **(A)** LVEF. **(B)** LVGLS in patients with acute myocarditis.

The ROC curve was drawn upon to determine cutoff values (Figure 3). A maximum LVEF of 36.46% indicated MACE with 75% sensitivity and 74.4% specificity (AUC: 0.774, *P*<0.001). Furthermore, a maximum LVGLS of 9% predicted MACE with 75% sensitivity and 73.5% specificity (AUC: 0.784, *P* < 0.001).

Based on the patient's electronic medical records, myocardial biopsy data were found in 11 (8.2%) cases. In two patients, the specimen was inadequate. Nine patients had evidence in favor of active myocarditis (myocardial inflammation and necrosis). The mean value and SD of the CMR parameters are depicted in Table 4. We assessed the MACE among these nine biopsy-proven patients and found three cases with ICD implantation, one with death, and three with heart transplantation. All patient had evidence of myocardial edema and fibrosis. LGE-VPS was evaluated, among them two patients had score five and nine, but others had score one.

**Table 4:** mean and SD of CMR parameters in nine myocarditis patients who underwent endomyocardial biopsy.

**Variables**	**Mean**	**SD (±)**
LGEVPS	2.81	2.48
LVEF	29.43	15.37
RVEF	36.23	16.09
LVEDVI	121.06	59.30
RVEDVI	97.83	34.06
LVGLS %	8.60	4.79
LVGCS %	10.28	6.40
RVGLS %	14.10	7.66
RVGCS %	7.32	5.27
LVGRS	20.62	16.88
RVGRS	12.61	9.62
LVGLS %	8.60	4.79
RSSR 1/sec	1.489	1.21
RDSR 1/sec	−1.50	0.95
CSSR 1/sec	−0.783	0.43
CDSR 1/sec	0.7418	0.42
LSSR 1/sec	−0.6245	0.36
LDSR 1/sec	0.8945	0.77

SD, standard deviation; LV, left ventricle; RV, right ventricle; LV, left ventricle; EF, ejection fraction; LA, left atrium; RA, right atrium; EDVI, end-diastolic volume index; ESVI, end-systolic volume index.

GLS, global longitudinal strain; GCS, global circumferential strain.

GRS, global radial strain; SV, stroke volume; LSSR, longitudinal systolic strain rat; CSSR, Circumferential systolic strain rate; RSSR, LSSR, Radial systolic strain rate; LDSR, longitudinal diastolic strain rate; CDSR, Circumferential diastolic strain rate; RDSR, Radial systolic strain.

## Discussion

This retrospective cohort analysis reviewed the CMR findings of 133 patients with the diagnosis of acute myocarditis referred for CMR at the first presentation. We assessed the relationship between cardiac prognosis and functional and FT-derived strain parameters. A summary of our study results is as follows:

1. The strongest predictors of MACE were LVEF and LVEDVI.
2. The ROC analysis showed a sensitivity of 75% and a specificity of 74% for a maximum LVEF of 36.5% in the prediction of MACE. Each 1-unit decrease in LVEF resulted in a 5% increase in the incidence of MACE.
3. Among deformation parameters, LVGLS was the most potent predictor of MACE. A maximum LVGLS of 9% could predict MACE with a sensitivity of 75% and a specificity of 74%. A 1% decline in LVGLS resulted in a 24% increase in the incidence of MACE.

In our study population, the incidence rate of MACE was about 12%. In a prospective analysis of 539 unselected patients with 37 cases of myocarditis performed by Mordi et al. MACE occurred in 8.1% over a mean follow-up time of 2.2 years. (7). In our investigation, we selected patients with the diagnosis of acute myocarditis who were positive for both myocardial edema and LGE in CMR images. We showed that basic CMR measurements, including LVEF and LVEDVI, were the most powerful predictors of MACE. These parameters could be simply derived in routine CMR examination and provide important prognostic information. Moreover, we found that the LGE extent estimated by VPS was not different between MACE positive and negative groups. Similarly, Sanguineti et al. showed that different CMR parameters, including myocardial inflammation and the extent of LGE, were not predictive of the outcome in patients with a CMR-derived diagnosis of myocarditis without severe hemodynamic compromise. However, the initial alteration of LVEF was the only independent CMR predictor of adverse clinical outcomes (19).

In the present investigation, we included LGE + patients with acute myocarditis and found that LV function played an essential role in predicting the outcome even in patients with morphologically altered myocarditis. Our results also showed that LVEF with a cutoff value of 36.5% was able to predict MACE. It is logical to assume that moderate LV systolic dysfunction is the strongest predictor of adverse cardiac events.

Our analysis of the correlation between strain parameters and adverse cardiac events revealed that LVGLS with a cutoff value of 9% was the strongest deformation parameter to predict mortality, ICD implantation, and heart transplantation within the follow-up. Similarly, in a study by Garcia-Ropero et al. FT-derived LVGLS was an independent predictor of prognosis and disease severity among patients with acute myocarditis (20). In another investigation by Vos et al. (21) unlike RV strain and LA functional values, LV deformation parameters were the independent predictors of prognosis in patients with acute myocarditis. Porcari et al. (22) demonstrated that LVGLS was an independent prognostic factor superior to LGE in acute myocarditis patients with an LVEF exceeding 50%.

It is assumed that GLS represents the function of longitudinally oriented subendocardial myocardial fibers (23), while GCS widely depicts the function of the mid-myocardial circumferential fibers. Different pathologies can affect subendocardial longitudinal and mid-myocardial myofibers. Fischer et al. analyzed the association between prognosis and the CMR features of 455 patients and the diagnosis of myocarditis during a median follow-up of 3.9 years. FT-derived LVGLS provided cumulative prognostic value over clinical features, LVEF, and LGE. Still, they found no association between cardiac events and LVGCS (6). However, we demonstrated that CMR parameters, including LVEF and LVEDVI, had a more predictive power than strains. It seems logical to presume that patients with worse LV function and dilated LV are the subgroups more prone to the presence of cardiac complications during follow-up. Among global LV strain parameters, GLS had the strongest predictive role for adverse outcomes, which could be because abnormalities within radial and circumferential myocardial fibers are common in patients with acute myocarditis, but damage to longitudinal myocardial fibers represents more severe involvement of the LV myocardium.

Interestingly, we found that each 1% decrease in absolute LVGLS value caused a 24% increase in the incidence of MACE. It confirms the meaningful role of minor LV longitudinal strain alterations in demonstrating hard cardiac events in patients with acute myocarditis. We believe that FT-CMR strain measurement will play a more prominent role in the management of patients with myocarditis in the future.

We divided the study population into two groups according to LVEF> or <40%. All global strain values, EF and LVEDVI, differed between the two groups. We noticed no difference in the LGE extent between the patients with and without MACE or LVEF> or <40%. This research investigated myocarditis patients with edema and LGE in the acute phase MRI, which may represent underlying inflammation to a great extent and not precisely reflect the fibrosis. Thus, we suppose that the initial LGE extent probably is not a potent predictor for adverse outcomes.

A dearth of follow-up information forced us to select only hard events (i.e., cardiac mortality, ICD implantation, and heart transplantation) as outcomes, making these prognostic factors more invaluable. Functional and FT strain parameters can be calculated without prescribing a gadolinium contrast agent or requiring a unique cardiac sequence. In addition, they can select patients with acute myocarditis who need intense follow-up visits.

Based on electronic medical records, we found eleven patients with cardiac biopsy results consisting of nine with evidence in favor of acute myocarditis. Interestingly, seven out of nine patients had MACE during follow-up. It may refer to selecting patients with a more acute nature of the disease for myocardial biopsy, but prospective studies focusing on the role of cardiac tissue sampling are required.

In this research, we encountered some limitations. Firstly, we had no access to prognosis in a minority of patients, prompting their exclusion from the study and reducing the sample size. Future larger-scale multicentric investigations could address this drawback. Secondly, a lack of imaging data in a significant number of the studied patients precluded us from employing novel mapping methods. Additionally, we reported a relatively higher incidence of MACE. We think it is somehow related to referral bias; Many low-risk young myocarditis patients do not refer for cardiac MRI. In our study, almost all events happened during the first 3 months after the acute event suggesting more severe cases. Further prospective studies are required to determine the exact incidence of MACE. Additionally, due to the retrospective design of our study, we could only access the limited data regarding the patient's history and risk profile, and for many other cardiac complications, we did not have a clear recording; therefore, we had to omit them from the study. Furthermore, we suppose that different experts' functional measurements, including strain values, and reporting the interobserver variability may improve the results' reliability. Last but not least, we included only patients with acute myocarditis; however, we suppose that a detailed CMR evaluation of other myocarditis categories and investigation of the prognostic role of measured parameters in each group will be of great value.

## Conclusions

The present study evaluated the role of functional and deformation parameters in predicting outcomes in patients with acute myocarditis. LVEF and LVEDVI had a significant role in indicating cardiac mortality, heart transplantation, and ICD implantation. In addition, LVGLS had a strong association with MACE among deformation parameters.

## Data availability statement

The raw data supporting the conclusions of this article will be made available by the authors without undue reservation.

## Ethics statement

The studies involving human participants were reviewed and approved by Rajaie Cardiovascular Medicine and Research Center. Written informed consent for participation was not required for this study in accordance with the national legislation and the institutional requirements.

## Author contributions

All authors listed have made a substantial, direct, and intellectual contribution to the work and approved it for publication.

## Conflict of interest

The authors declare that the research was conducted in the absence of any commercial or financial relationships that could be construed as a potential conflict of interest.

## Publisher's note

All claims expressed in this article are solely those of the authors and do not necessarily represent those of their affiliated organizations, or those of the publisher, the editors and the reviewers. Any product that may be evaluated in this article, or claim that may be made by its manufacturer, is not guaranteed or endorsed by the publisher.
